# The causal correlation between gut microbiota abundance and pathogenesis of cervical cancer: a bidirectional mendelian randomization study

**DOI:** 10.3389/fmicb.2024.1336101

**Published:** 2024-02-14

**Authors:** Hua Yang

**Affiliations:** Department of Gynecology, The Fifth Affiliated Hospital of Sun Yat-sen University, Zhuhai, China

**Keywords:** cervical cancer, causal relationship, genome-wide association studies, gut microbiota, Mendelian randomization

## Abstract

**Background:**

Observational studies and animal experiments suggested potential relevance between gut microbiota (GM) and cervical cancer (CC), but the relevance of this association remains to be clarified.

**Methods:**

We performed a two-sample bidirectional Mendelian randomization (MR) analysis to explore whether there was a causal correlation between GM and CC, and the direction of causality.

**Results:**

In primary outcomes, we found that a higher abundance of *class Clostridia, family Family XI, genus Alloprevotella, genus Ruminiclostridium 9,* and *order Clostridiales* predicted higher risk of CC, and a higher abundance of *class Lentisphaeria, family Acidaminococcaceae, genus Christensenellaceae R7 group, genus Marvinbryantia, order Victivallales, phylum Actinobacteria,* and *phylum Lentisphaerae* predicted lower risk of CC. During verifiable outcomes, we found that a higher abundance of *class Methanobacteria, family Actinomycetaceae, family Methanobacteriaceae, genus Lachnospiraceae UCG 010, genus Methanobrevibacter, order Actinomycetales,* and *order Methanobacteriales* predicted a higher risk of CC, and a higher abundance of *family Streptococcaceae, genus Dialister,* and *phylum Bacteroidetes* predicted a lower risk of CC, and vice versa.

**Conclusion:**

Our study implied a mutual causality between GM and CC, which provided a novel concept for the occurrence and development of CC, and might promote future functional or clinical analysis.

## 1 Introduction

The incidence of cervical cancer (CC) is only lower than that of breast, lung, and colorectal cancers in women worldwide. It has the highest incidence of malignant tumors in the female reproductive tract. Nearly 530,000 new CCs are diagnosed worldwide each year, causing serious health and economic burdens in both developing and developed countries (He and Li, 2021). The etiological effect of high-risk human papillomavirus (hr-HPV) has been established for decades and it has been found in over 99.7% of women with CC. However, hr-HPV infection is very common in sexually active women, and the incidence of CC is relatively low. Over 90% of hr-HPV infections regress naturally (Viveros-Carreño et al., 2023). Infection with hr-HPV is essential but insufficient for the pathogenesis of CC, and additional factors, such as immune factors and vaginal microecology, may play a role in the occurrence and progression of CC.

CC is regarded as a multifactorial disease, the mechanism and process of carcinogenesis are largely unknown and may involve several environmental, lifestyle, and hereditary factors, such as sexual behavior, parity, use of hormonal contraceptives, individual immunity, and smoking (Zhang et al., 2021). The human digestive tract carries over 1,000 microbes defined as the gut microbiota (GM). It is essential for health and plays a role in multiple physiological processes, including metabolism, detoxification, nutrient absorption, maintenance of homeostasis of the intestinal mucous barrier, and the immune and endocrine systems. Furthermore, the alteration of the GM, such as altered composition and abundance, may cause damage to the mucosal barrier, translocation of bacteria and endotoxins, may cause a variety of inflammation, might compromise the immune environment, may change the metabolome, and so on. Recent research has found that alterations in the GM were closely associated with a variety of tumors in and outside the gut tract, such as liver cancer, ovarian cancer, colorectal cancer, pancreatic cancer, and breast cancer (Yu and Schwabe, 2017; Ma et al., 2018; Chen et al., 2019; Parida and Sharma, 2019; Plaza-Díaz et al., 2019).

Several studies have reported a potential association between CC and GM. Karpinets et al. (2020) did not identify any relationship between GM alteration caused by oral antibiotics and CC development. Wang et al. (2019) compared the GM profiles in eight CC patients with five healthy controls and found an increased alpha diversity and clear separation in beta diversity in CC-associated gut microbiota. Kang et al. (2020) compared the fecal microbiota of 17 early CC patients to that of 29 healthy women and found a significant difference in Chao1 diversity between the two groups, with consistent outcomes in observed operational taxonomic unit analysis, and a prediction model based on fecal analysis was helpful for early diagnosis of CC. Chang et al. (2023) compared the microbiota profiles in fecal samples of 13 CC patients with 10 healthy controls and found a significant difference in GM abundance between women with CC and healthy controls. They also found that the abundance of *Ruminococcus 2* was negatively correlated with the CC stage. Sims et al. (2019) compared the microbiota profiles in fecal samples of 42 CC with 46 healthy controls, found increased alpha diversity and beta diversity in the women with CC and the abundance of *Dialister, Prevotella, and Porphyromonas* were significantly higher in the women with CC, while the abundance of *Lachnospiracea, Bacteroides*, and *Alistipes* was significantly higher in healthy women. Although these studies suggest that the gut microbiota is correlated with CC, the real effect and impact on CC are largely unknown. The causal relationship between gut microbiota and CC has been insufficiently addressed due to the limitations of conventional observational studies, which are susceptible to potential confounding bias or reverse causal bias.

Mendelian randomization (MR) analysis is an epidemiological statistical method that can overcome the limitations of traditional observational studies and may avoid the bias of confounding factors or reverse causality (Bowden and Holmes, 2019) because it adopts germline randomly assigned single nucleotide polymorphisms (SNPs) to compute the causal correlation degree between exposures and outcomes. To explore the causal role and direction of the causal correlation between gut microbiota abundance and CC, we employed a two-sample bidirectional MR analysis during the present study.

## 2 Materials and methods

### 2.1 GWAS (Genome-Wide Association Studies) statistics of CC

This study enrolled two public CC datasets. The first, a GWAS (ID: ukb-b-8777; Elsworth et al., 2013; (Cancer code, self-reported: cervical cancer)), enrolled 1,889 CC patients and 461,044 non-gender-specific health controls from the European population. The second GWAS (ID: ieu-b-4876; Hemani et al., 2018) enrolled 563 CC patients, and the 198,523 non-sex-specific health controls came from the European population.

### 2.2 GWAS statistics of gut microbiota abundance

The GWAS data from a study (ID: ebi-a-GCST90017108) on GM abundance (Wang et al., 2018) was published in 2021 and included a 14,306 sample size from the European population. The data were coordinated with gene sequencing profiles based on 16S ribosomal RNA, and a total of 197 taxa (9 phyla, 16 classes, 19 orders, 33 families, and 120 genera) were included, and 14 unknown taxa (11 genera and 3 families) were excluded.

### 2.3 Instrumental variable selection

GM abundance was comprehensively analyzed in distinct independent taxa. To ensure the robustness and veracity of the analysis results, the following optimization strategies were adopted to extract closely related independent variables (IVs). First, we set a strong statistical threshold of *p* < 5 × 10^−8^ to extract SNPs intensively correlated with GM abundance, which was regarded as a conventional genome-wide significance level. Unfortunately, no SNPs were extracted from most taxa of the gut microbiota. We used the second threshold of *p* < 5 × 10^−6^ for the MR analysis, which was based on previous literature. Second, we set the threshold for the minor allele frequency (MAF) to 0.01 to filter common spontaneous SNP mutations. Third, the key rules of the MR analysis excluded the bias caused by linkage disequilibrium (LD) among IVs (Kurilshikov et al., 2021). In the present study, we set R^2^ < 0.001 and clumping distance = 10,000 kb as a threshold to clump SNPs with LD. Fourth, to ensure that the effectiveness of the SNPs on exposure corresponded to the same allele on outcome, we clamped palindromic SNPs to avoid the substitution of strand directionality or allele coding.

The horizontal pleiotropy of the SNPs was tested using MR-PRESSO (Li et al., 2022). The MR-PRESSO outlier trial was used to compute the value of *p* for single significant pleiotropy, whereas the global trial was used to compute the value of *p* for overall significant pleiotropy. SNPs were arranged in increasing order of *p*-values and then removed one by one. The MR-PRESSO global trial was adopted to compute the value of *p* for the remaining SNPs again until *p* > 0.05. The remaining SNPs were used for subsequent MR analysis.

### 2.4 Mendelian randomization statistical analysis

A bidirectional two-sample MR was used to infer the causal correlation between GM abundance and CC. To test whether GM abundance was causally affected by CC, we selected SNPs that were closely related to GM abundance. GWAS data: ukb-b-8777 was set as the primary outcome and GWAS data: ieu-b-4876 was set as the verified outcome. To test whether CC altered GM abundance, SNPs closely related to CC were selected as exposure in the reverse MR analysis process, with GM abundance as the outcome.

Three mainstream MR methods were adopted for multiple SNPs MR analysis: inverse-variance weighted (IVW), weighted median estimator (WME), and MR-Egger regression (Bowden et al., 2015). The IVW method was regarded as more robust than the WME and MR-Egger regressions. Therefore, the MR results mainly depended on the IVW method. The Wald ratio test was used to evaluate the association between the gut microbiota taxa and CC for only one SNP.

Several sensitivity tests were conducted to assess the reliability of the results. Leave-one-out test (Gnona and Stewart, 2022) was used to assess whether the causal correlation was caused by a single SNP. A causal direction test was used to compare the variance caused by the SNPs in the exposure to the outcome. If the SNPs caused greater variance in exposure than in the outcome, causality was known as directional robustness. F-statistics were calculated to avoid a weak IV bias (Cheng et al., 2017). *F*-values < 10 were defined as weak IVs and were excluded from the subsequent MR analysis.

All analyses were performed using R for Windows, version 4.3.0. The “TwoSampleMR” package and “MR-PRESSO” package were adopted.

### 2.5 Heterogeneity

Cochran’s Q statistic was used for the heterogeneity test (Burgess and Thompson, 2011). A Q value > number of SNPs −1 or a value of *p* < 0.05 suggested heterogeneous and invalid IVs.

**Figure 1:**
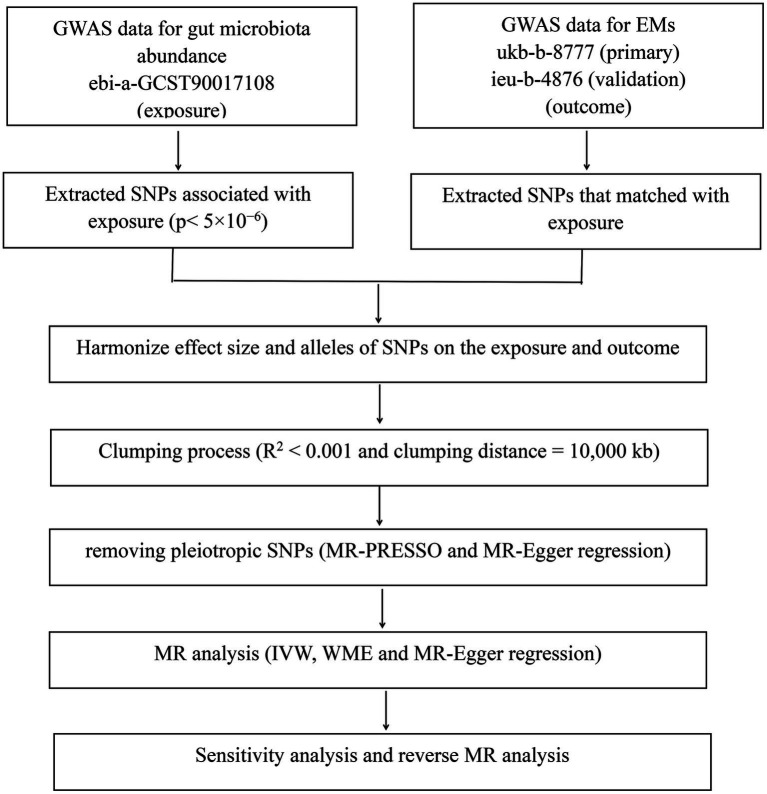
The flowchart of the present MR analysis.

The flowchart of the present MR analysis is presented in Figure 1.

## 3 Results

### 3.1 SNP selection

First, we extracted 1 to 11 SNPs associated with single GM taxa for a total of 183 taxa (8 phyla, 16 classes, 20 orders, 29 families, and 110 genera) at a significance threshold of *p* < 5 × 10^−6^ according to the aforementioned optimization strategies. The number of SNPs in each taxon is detailed in Supplementary Table S1. The *F*-value of each SNP was greater than 10, indicating that no weak IVs basis existed, and no pleiotropic effects were identified by the MR-PRESSO global trial (*p* > 0.05).

### 3.2 Primary causality of GM abundance on the risk of CC

**Figure 2:**
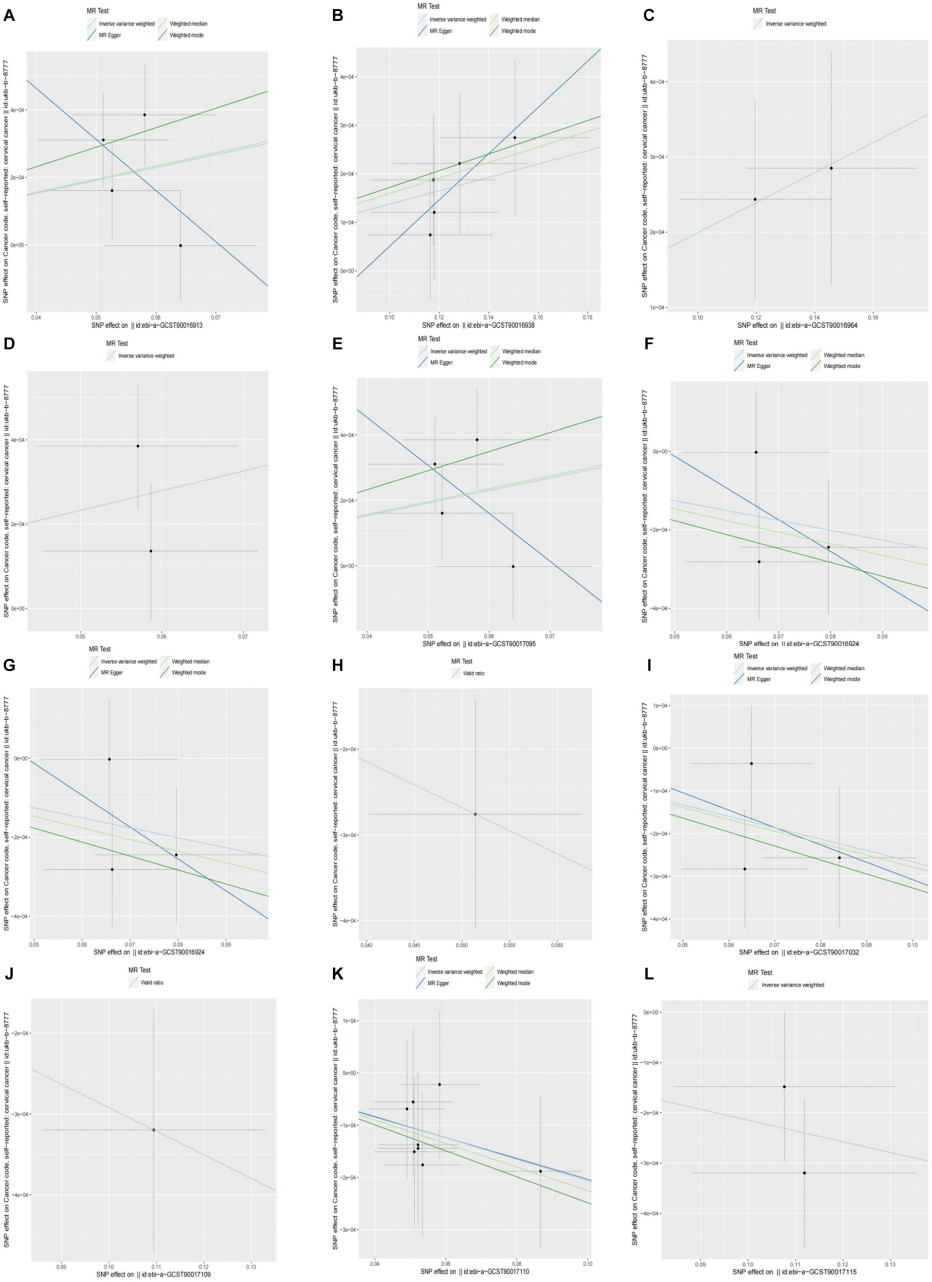
Causality of GM abundance on the risk of CC from GWAS data: ukb-b-8777. **(A)** the method comparison of MR results between *class Clostridia* and CC. **(B)** The method comparison of MR results between *family Family XI* and CC. **(C)** The method comparison of MR results between *genus Alloprevotella* and CC. **(D)** The method comparison of MR results between *genus Ruminiclostridium 9* and CC. **(E)** The method comparison of MR results between *order Clostridiales* and CC. **(F)** The method comparison of MR results between *family Acidaminococcaceae* and CC. **(G)** the method comparison of MR results between *class Lentisphaeria* and CC. **(H)** The method comparison of MR results between *genus Christensenellaceae R7 group* and CC. **(I)** The method comparison of MR results between *genus Marvinbryantia* and CC. **(J)** the method comparison of MR results between *order Victivallales* and CC. **(K)** the method comparison of MR results between *phylum Actinobacteria* and CC. **(L)** the method comparison of MR results between *phylum Lentisphaerae* and CC.

When setting the statistical threshold as *p* < 5 × 10^−6^ and GWAS data: ukb-b-8777 as the outcome, we found that a higher abundance of *class Clostridia* causally predicted a higher risk of CC (b = 0.00382, *p* = 0.01526 by IVW test), with homogenous results by MR Egger and weighted median test, no horizontal polymorphism (*p* = 0.385), and heterogeneity (*p* = 0.4014) were found between SNPs. Causal direction analysis found that the variance explained in exposure was significantly stronger than the variance explained in outcome (*p* = 4.34 e-36), and the leave-one-out test found that causality was not affected by a single SNP. The method comparison of the MR results is plotted in Figure 2A, which suggests that the causal correlation between *class Clostridia* and CC was robust. We also found that a higher abundance of *family Family XI, genus Alloprevotella, genus Ruminiclostridium 9,* and *order Clostridiales* causally predicted a higher risk of CC (Supplementary Table S2; Figures 2B–E). Meanwhile, we found that a higher abundance of *family Acidaminococcaceae* causally predicted a lower risk of CC (b = −0.002515, *p* = 0.03575 by IVW test), with homogenous results by MR Egger and weighted median test, no horizontal polymorphism (*p* = 0.819), and heterogeneity (*p* = 0.4002) found between SNPs. Causal direction analysis found that the variance explained in exposure was significantly stronger than the variance explained in outcome (*p* = 1.4e-05), and the leave-one-out test found that causality was not affected by a single SNP. The method comparison of MR results is plotted in Figure 2F, which suggests that the causal correlation between *family Acidaminococcaceae* and CC was robust. We also found a higher abundance of *class Lentisphaeria, genus Christensenellaceae R7 group, genus Marvinbryantia, order Victivallales, phylum Actinobacteria,* and *phylum Lentisphaerae* (Supplementary Table S2; Figures 2G–L).

### 3.3 Verified causality of GM abundance on the risk of CC

**Figure 3:**
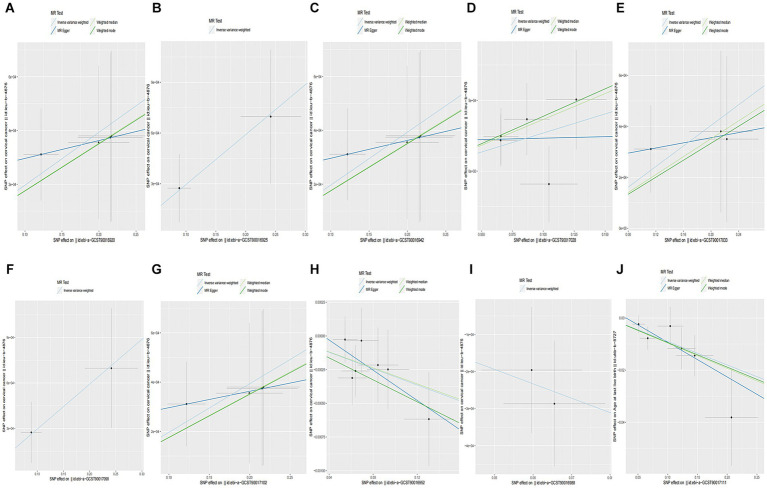
Causality of GM abundance on the risk of CC from GWAS data: ieu-b-4876. **(A)** The method comparison of MR results between *class Methanobacteria* and CC. **(B)** The method comparison of MR results between *family Actinomycetaceae* and CC. **(C)** The method comparison of MR results between *family Methanobacteriaceae* and CC. **(D)** The method comparison of MR results between *genus Lachnospiraceae UCG 010* and CC. **(E)** The method comparison of MR results between *genus Methanobrevibacter* and CC. **(F)** The method comparison of MR results between *order Actinomycetales* and CC. **(G)** The method comparison of MR results between *order Methanobacteriales* and CC. **(H)** The method comparison of MR results between *family Streptococcaceae* and CC. **(I)** The method comparison of MR results between *genus Dialister* and CC. **(J)** The method comparison of MR results between *phylum Bacteroidetes* and CC.

**Figure 4:**
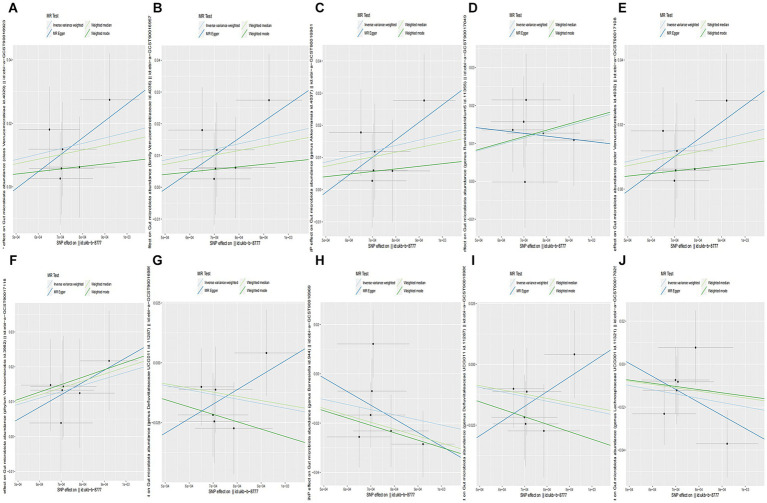
Causality of CC from GWAS data: ukb-b-8777 on GM abundance. **(A)** The method comparison of MR results between CC and *class Verrucomicrobiae*. **(B)** The method comparison of MR results between CC and *family Verrucomicrobiaceae*. **(C)** The method comparison of MR results between CC and *genus Akkermansia*. **(D)** The method comparison of MR results between CC and *genus Ruminiclostridium5*. **(E)** The method comparison of MR results between CC and *order Verrucomicrobiales*. **(F)** The method comparison of MR results between CC and *phylum Verrucomicrobia*. **(G)** The method comparison of MR results between CC and *family Defluviitaleaceae*. **(H)** The method comparison of MR results between CC and *genus Barnesiella*. **(I)** The method comparison of MR results between CC and *genus Defluviitaleaceae UCG011*. **(J)** The method comparison of MR results between CC and *genus Lachnospiraceae UCG001*.

When setting the statistical threshold as *p* < 5 × 10^−6^ and GWAS data: ieu-b-4876 as the outcome, we found that a higher abundance of *class Methanobacteria* causally predicted a higher risk of CC (b = 0.001967, *p* = 0.01526 by IVW test), with homogenous results by MR Egger and weighted median test. No horizontal polymorphism (*p* = 0.673), and heterogeneity (*p* = 0.9709) were found between SNPs, with insufficient data for causal direction analysis, and the leave-one-out test found that causality was not affected by a single SNP. Causal direction analysis found that the variance explained in exposure was significantly stronger than the variance explained in the outcome (*p* = 2.79e-18). The method comparison of MR results was plotted in Figure 3A, which suggested that the causal correlation between *class Methanobacteria* and CC was robust. We also found that a higher abundance of *family Actinomycetaceae, family Methanobacteriaceae, genus Lachnospiraceae UCG 010, genus Methanobrevibacter, order Actinomycetales,* and *order Methanobacteriales* causally predicted a higher risk of CC (Supplementary Table S3; Figures 3B–G). Meanwhile, we found that a higher abundance of *family Streptococcaceae* causally predicted a lower risk of CC (b = −0.003037, *p* = 0.001172 by IVW test), with homogenous results by MR Egger and weighted median test, no horizontal polymorphism (*p* = 0.574), and heterogeneity (*p* = 0.9043) between SNPs. The causal direction analysis found that the variance explained in the exposure was significantly stronger than that in the outcome (*p* = 1.27e-40). The leave-one-out test revealed that causality was not affected by a single SNP. The method comparison of the MR results is plotted in Figure 4H, which suggests that the causal correlation between *family Streptococcaceae* and CC was robust. We also found that a higher abundance of *genus Dialister* and *phylum Bacteroidetes* causally predicted a lower risk of CC (Supplementary Table S3; Figures 3I,J).

### 3.4 Primary causality of CC on GM abundance

When the statistical threshold was set at *p* < 5 × 10^−6^, seven closely related SNPs were extracted as IVs for GWAS data: ukb-b-8777 gut microbiota taxa as the outcome. We found that CC causally predicted a higher abundance of *class Verrucomicrobiae* (b = 17.12, *p* = 0.03145 by IVW test), with homogenous results by the MR Egger and weighted median test. No horizontal polymorphism (*p* = 0.612), and heterogeneity (*p* = 0.8866) were found between SNPs. The leave-one-out test revealed that causality was not affected by a single SNP. A comparison of the MR results is plotted in Figure 4A, which suggests that the causal correlation between CC and *class Verrucomicrobiae* was robust. However, causal direction analysis found that the variance explained in exposure was not significantly different from the outcome (*p* = 0.928), which meant that the reverse causal relationship could not be excluded. We also found that CC causally predicted a higher abundance of *family Verrucomicrobiaceae, genus Akkermansia, genus Ruminiclostridium5, order Verrucomicrobiales,* and *phylum Verrucomicrobia* (Supplementary Table S4; Figures 4B–F). Furthermore, we found that CC causally predicted a lower abundance of *family Defluviitaleaceae* (b = −20.5, *p* = 0.03323 by IVW test), with homogenous results by MR Egger and weighted median test, no horizontal polymorphism (*p* = 0.469), and heterogeneity (*p* = 0.843) between SNPs, and the leave-one-out analysis found that the causality was not affected by a single SNP. The method comparison of MR results is plotted in Figure 4G, which suggests that the causal association between CC and *family Defluviitaleaceae* was robust; however, the causal direction analysis found that the variance explained in exposure was insignificantly different from the outcome (*p* = 0.605), which meant that the reverse causal relationship could not be excluded. We also found that CC causally predicted a lower abundance of *genera Barnesiella, Defluviitaleaceae UCG011,* and *Lachnospiraceae UCG001* (Supplementary Table S4; Figures 4H–J).

### 3.5 Verified causality of CC on GM abundance

When the statistical threshold was set as *p* < 5 × 10^−6^, 14 closely related SNPs were extracted as IVs for GWAS data: ieu-b-4876, GM taxa as the outcome. We found that CC causally predicted a lower abundance of *genus Alloprevotella* (b = −0.1618, *p* = −0.1618 by IVW test), with homogenous results by the MR Egger and weighted median test. No horizontal polymorphism (*p* = 0.526), and heterogeneity (*p* = 0.1685) were found between SNPs, and the leave-one-out test found that causality was not affected by a single SNP. The method comparison of MR results is plotted in Supplementary Figure S1A. These results suggested that the causal correlation between CC and *genus Alloprevotella* was robust. However, there were not enough SNPs for causal direction analysis, so a reverse causal relationship could not be excluded. We also found that CC causally predicted a lower abundance of *genus Eubacteriumnodatum group* and *genus Phascolarctobacterium* (Supplementary Table S5; Supplementary Figures S1B,C). Furthermore, we found that CC causally predicted a higher abundance of *genus Eisenbergiella* (b = 22.22, *p* = 0.03636 by IVW test), with homogenous results by MR Egger and Weighted median test. No horizontal polymorphism (*p* = 0.94), and heterogeneity (*p* = 0.9326) were found between SNPs, and the leave-one-out analysis found that the causality was not affected by a single SNP. The method comparison of MR results is plotted in Supplementary Figure S1D, which suggests that the causal association between CC and *genus Eisenbergiella* was robust. However, the causal direction test found that the variance explained in the exposure was not significantly different from the outcome (*p* = 0.382), which meant that the reverse causal relationship could not be excluded. We also found that CC causally predicted a higher abundance of *phylum Euryarchaeota* (Supplementary Table S5; Supplementary Figure S1E).

## 4 Discussion

To the best of our knowledge, the present study was an ingenious MR study to explore the causal correlation between GM abundance and CC. We thought it had important clinical practice guiding significance for microbiome and CC studies. Robustly associated SNPs were extracted from the largest GWAS for GM abundance and two independent CC databases. According to the comprehensive genetic correlation analysis of over 670,000 European individuals, we found that SNPs’ predisposition to some GM taxa had a causal relationship with CC; furthermore, we also found that SNPs’ predisposition to CC had a causal relationship with some GM taxa. These results have implications for a novel direction for non-invasive early diagnosis of CC; further, the GM might be a novel target for prevention, treatment, and long-term management of CC.

CC is the most common gynecological neoplasia in developing countries and poses a serious health threat to women worldwide. Although hr-HPV infection is a direct etiological factor of CC, the mechanism of carcinogenesis is largely unknown. Nearly 85%–90% of hr-HPV infections are spontaneously resolved, and only 10%–15% remain, which might cause precancerous neoplasia and further progress to CC. In the past few years, owing to the rapid development of science technologies, omics research, bioinformatics, and high-throughput sequencing technology, a growing body of research has found that the vaginal micro-ecosystem plays a key role in the progression from hr-HPV infection to CC (Sanderson et al., 2019; Łaniewski et al., 2020; Castanheira et al., 2021; Kyrgiou and Moscicki, 2022). Given the close relationship between the endovaginal and gut microbiome through bacterial movement and colonization between the genital and gastrointestinal tracts, we speculated that GM might be involved in the carcinogenesis of CC. Indeed, several studies have found a potential association between CC and GM, but the results were inconsistent as to whether there was a causal correlation, and the direction of the causal correlation between GM and CC was still unclear.

In this MR study, dual verification was adopted to verify the robustness of causality. For the primary analysis, we used GWAS data:ukb-b-8777 as the outcome and found genetic liability to *class Clostridia, class Lentisphaeria, family Acidaminococcaceae, genus Butyricicoccus, Family XI, genus Alloprevotellagenus, genus Christensenellaceae R7 group, genus Marvinbryantia, genus Ruminiclostridium 9, order Clostridiales, order Victivallales, phylum Actinobacteria,* and *phylum Lentisphaerae* was causally associated with CC. For verifiable analysis, we set GWAS data: lieu-b-4876 as the outcome and found genetic liability to *class Methanobacteria, family Actinomycetaceae, family Methanobacteriaceae, family Streptococcaceae, genus Dialister, genus Lachnospiraceae UCG 010, genus Methanobrevibacter, order Methanobacteriales,* and *phylum Bacteroidetes* was causally associated with CC. Our results suggest that certain gut microbiota taxa might be involved in CC pathogenesis, and GM analysis might help identify females at high risk for CC and might help in the early diagnosis of CC at an earlier time.

Until now, the mechanism by which GM affects CC has been largely unknown. One hypothesis was that GM might activate Toll-like receptors (TLRs; Chang et al., 2020) and pro-inflammatory cytokines such as interleukin-17 (IL-17; Brevi et al., 2020) and tumor necrosis factor (TNF; Piñero et al., 2019), ultimately leading to carcinogenesis. Indeed, *in vivo* and *in vitro* studies have shown that TLRs are key mediators in bacteria-triggered cancer. Lipopolysaccharide (LPS) derived from GM could induce hepatocellular carcinoma by activating TLR4 in immune cells (Liu et al., 2022). Similarly, Ochi et al. (2012) found that TLR4 was a key factor mediating carcinogenesis from pancreatic inflammatory disease to pancreatic cancer. Several studies in recent decades have found obvious alterations in the GM composition of women with CC; for example, Wang et al. (2019) found that the abundance of Bacteroidetes was significantly upregulated in fecal specimens of women with CC, which was confirmed by our Mendelian randomization study.

Microbial enterovaginal transfer might be another potential mechanism. Persistent infection with hr-HPV and CC are directly linked to abnormal vaginal microbiota. Alterations in the vaginal microbiome affect the risk of human papillomavirus (HPV) infection and persistence, further affecting CC risk. The sharing between genital and gastrointestinal tracts was confirmed by clinical observation and experimental studies and might be mediated by motility and colonization of the vagina from fecal pellets. Transfer is regarded as the main way to improve the diversity of the vaginal microbiome. Karpinets et al. (2020) found that antibiotics increased the abundance of the vaginal microbiome, further lowering the risk of CC in a murine model. Ritu et al. (2019) explored the relationship between cervical microbiota abundance and HPV infection in healthy women and found that *genus Dialister* was closely related to poor HPV status, including newly acquired and persistent infection. Our study also found that the genetic liability to *genus Dialister* was causally associated with CC. These coupled results suggest the rational pathogenicity of *genus Dialister* for CC.

Although HPV could be considered as the primary cause of almost all CC, growing evidence suggests that the prevalence of HPV-negative patients is not negligible, which might be the result of alternative pathways such as the TP53—related pathway, nuclear factor kappa B (NF-kB) pathway, reactive oxygen species (ROS), or free radicals during vaginal microenvironment (Giuliano, 2003), which are closely related with the vaginal and gut microbiota. These might be the possible mechanisms that mediate the causal relationship between GM and CC.

*In vivo* studies by Chung and Lambert (2009) suggested that estrogen might be involved in the carcinogenesis of CC. Epidemiological data (Chung et al., 2010; Bronowicka-Kłys et al., 2016; Yu et al., 2018) also confirmed that women with the highest serum estrogen levels had an increased risk of CC. Therefore, estrogen might be another potential mechanism mediated by the gut microbiota affecting CC. Previous research has shown that alterations in the gut microbiota might lead to increased circulatory estrogen levels. Certain taxa gut microbiota could produce β-glucuronidase or β-glucosidases involved in estrogen metabolism, which is defined as the “estrobolome” (Baker et al., 2017; Ervin et al., 2019; Hu et al., 2023). Estrogen metabolism mainly occurs in the liver. The liver can inactivate estrogen through sex hormone-binding globulin. β-glucuronidase or β-glucosidases originate from the gut microbiota and catalyze the degradation of conjugated estrogen, and the reabsorption of estrogen from the intestine increases. High-throughput sequencing of the gut microbial genome revealed that multiple bacterial taxa carried the gene coded for β-glucuronidase or β-glucosidases, including *Bacteroides, Bifidobacterium, Escherichia,* and *Lactobacillus.* Indeed, during our MR study, we found genetic liability to *phylum Bacteroidetes* (belonging to the estrobolome) was causally associated with CC, suggesting that GM might be involved in the pathogenesis of CC through estrogen metabolism.

Although numerous clinical studies have reported that GM abundance was significantly different between CC and healthy females, the results were inconsistent. Whether the gut microbiota abundance changed before and after the onset of CC in the same female has not yet been clarified. Whether CC could cause alterations in gut microbiota abundance was unknown, which seemed to be difficult to solve by epidemiological or observational studies. Therefore, we adopted a reverse MR study to clarify this puzzle.

During the reverse MR study, we set GWAS data: ukb-b-8777 as exposure, and MR results found genetic liability to CC was causally associated with *class Verrucomicrobiae, family Defluviitaleaceae, family Verrucomicrobiaceae, genus Akkermansia, genus Barnesiella, genus Defluviitaleaceae UCG011, genus Lachnospiraceae UCG001, genus Ruminiclostridium5, order Verrucomicrobiales,* and *order Verrucomicrobiales*. For verifiable analysis, we set GWAS data: lieu-b-4876 as exposure, and MR results found genetic liability to CC was causally associated with *genera Alloprevotella, Eisenbergiella, Eubacterium nodatum, Phascolarctobacterium,* and *Euryarchaeota.* Our results suggested that CC might affect certain GM taxa, which means that GM analysis might be a novel target for non-invasive diagnosis of CC. However, the exact process by which CC affects GM is largely unknown, which is a crucial implication for further research.

The main strengths of our research included the ingenious MR analysis of the causality between GM abundance and CC, enrollment of the largest sample sizes until now, and dual verification to verify the robustness of the results. MR analysis eliminated the confounding bias that is inevitable in epidemiological studies, which had similar levels of evidence as randomized controlled trials (RCT). Moreover, our SNPs were strongly associated with GM and were compared using two dependent CC databases. Moreover, the sensitivity analysis showed no pleiotropy or heterogeneity, indicating that our results were statistically robust.

Nevertheless, our study had several limitations. First, the populations in the GWAS data used in our study were European, and, as ethnic and geographical factors might affect GM abundance, this might lead to limited effects extending to other populations. Second, owing to the summary data, individual characteristics were unavailable, and the confounding bias of individualized features was inestimable. Third, due to our strict thresholds, many of the genetic liabilities of GM abundance were excluded at the IV selection stage, which might have resulted in missing some meaningful results.

Future research should enroll larger samples from multiple races and geographic areas to explore more robust causality. Furthermore, more in-depth mechanistic research is urgently needed, and the diagnostic and therapeutic value of targeting GM abundance in CC requires further research.

## 5 Conclusion

We comprehensively assessed the relationship between GM abundance and CC. Our results suggested that 22 GM taxa were causally related to CC, while CC was causally related to 15 GM taxa. Our study implies a mutual causality between GM abundance and the pathogenesis of CC, which provides a novel concept for the occurrence and development of CC and might promote future functional or clinical analysis.

## Data availability statement

The original contributions presented in the study are included in the article/Supplementary material, further inquiries can be directed to the corresponding author.

## Author contributions

HY: Conceptualization, Data curation, Investigation, Methodology, Software, Supervision, Writing – original draft, Writing – review & editing.
