# Supplementary material for: The causal correlation between gut microbiota abundance and pathogenesis of cervical cancer: a bidirectional mendelian randomization study
Source: Front Microbiol. 2024 Feb 14;15:1336101. doi: 10.3389/fmicb.2024.1336101 (PMC10901247; doi:10.3389/fmicb.2024.1336101)
Supplement: Supplementary file 1 [file Table_1.docx]

Table S1. The detailed numbers of SNPs and IVW/Wald ratio results for each taxon

| Gut microbiota | n SNP | IVW/Wald ratio | | |
| --- | --- | --- | --- | --- |
|  |  | b value | SE | P value |
| order Verrucomicrobiales | 6 | 0.0001352 | 0.001128 | 0.9046 |
| phylum Euryarchaeota | 2 | 0.001264 | 0.0008385 | 0.1317 |
| genus Fusicatenibacter | 3 | 0.0005697 | 0.001891 | 0.7632 |
| family Bifidobacteriaceae | 4 | 0.0006141 | 0.002156 | 0.7758 |
| genus Peptococcus | 4 | -0.0003534 | 0.000718 | 0.6226 |
| genus Eubacterium fissicatena group | 4 | -0.0002525 | 0.0006002 | 0.674 |
| family Oxalobacteraceae | 4 | 0.0000779 | 0.001126 | 0.9448 |
| genus Ruminococcus1 | 2 | -0.0001045 | 0.001808 | 0.9539 |
| genus Ruminococcus2 | 3 | 0.00001391 | 0.001401 | 0.9921 |
| family Victivallaceae | 4 | -0.0002582 | 0.0008837 | 0.7701 |
| genus Desulfovibrio | 3 | 0.001266 | 0.001199 | 0.2912 |
| genus Anaerofilum | 4 | 0.00006582 | 0.0008601 | 0.939 |
| order Bifidobacteriales | 4 | 0.0006141 | 0.002156 | 0.7758 |
| genus Ruminococcaceae UCG010 | 4 | -0.0002785 | 0.001122 | 0.804 |
| class Clostridia | 4 | 0.00382 | 0.001575 | 0.01526 |
| genus Adlercreutzia | 2 | -0.0006902 | 0.001261 | 0.584 |
| genus Lachnospiraceae FCS020 group | 4 | 0.000472 | 0.001835 | 0.797 |
| genus Lachnospiraceae UCG008 | 5 | 0.0001317 | 0.0009025 | 0.884 |
| class Melainabacteria | 2 | 0.0002119 | 0.001036 | 0.838 |
| phylum Firmicutes | 4 | -0.0004057 | 0.00134 | 0.7621 |
| genus Clostridium innocuum group | 1 | -0.00186 | 0.001243 | 0.1346 |
| genus Eubacterium rectale group | 3 | 0.0004243 | 0.002913 | 0.8842 |
| class Bacilli | 5 | -0.00001596 | 0.001613 | 0.9921 |
| family Verrucomicrobiaceae | 6 | 0.0001356 | 0.001129 | 0.9044 |
| family Bacteroidales S24 7group | 4 | 0.0002209 | 0.001074 | 0.8371 |
| order Burkholderiales | 5 | 0.001107 | 0.00124 | 0.3721 |
| genus Catenibacterium | 3 | -0.0006247 | 0.0006512 | 0.3374 |
| phylum Lentisphaerae | 2 | -0.002147 | 0.0009549 | 0.02456 |
| family Peptococcaceae | 4 | -0.000967 | 0.001005 | 0.3358 |
| genus Marvinbryantia | 3 | -0.002662 | 0.001197 | 0.02618 |
| genus Butyrivibrio | 4 | -0.0001715 | 0.0008366 | 0.8376 |
| genus Eisenbergiella | 4 | -0.0005035 | 0.0007507 | 0.5024 |
| genus Defluviitaleaceae UCG011 | 3 | -0.0003113 | 0.001035 | 0.7635 |
| genus Ruminococcaceae UCG003 | 5 | 0.0004005 | 0.001049 | 0.7027 |
| phylum Verrucomicrobia | 4 | -0.0003756 | 0.001601 | 0.8146 |
| genus Ruminococcaceae UCG005 | 4 | -0.003061 | 0.001526 | 0.04485 |
| genus Ruminococcus gnavus group | 3 | 0.0003399 | 0.0008984 | 0.7051 |
| genus Dialister | 2 | -0.002821 | 0.002153 | 0.1901 |
| genus Gordonibacter | 1 | -0.0006375 | 0.001132 | 0.5733 |
| genus Anaerostipes | 6 | -0.00006024 | 0.001071 | 0.9551 |
| class Lentisphaeria | 1 | -0.002922 | 0.001354 | 0.03095 |
| family Enterobacteriaceae | 2 | 0.0003297 | 0.001657 | 0.8422 |
| order Lactobacillales | 3 | 0.001072 | 0.00255 | 0.6742 |
| genus Ruminococcaceae UCG004 | 2 | -0.0004555 | 0.001156 | 0.7243 |
| order Mollicutes RF9 | 1 | -0.002117 | 0.002017 | 0.2941 |
| family Lachnospiraceae | 6 | 0.00008719 | 0.001205 | 0.9423 |
| genus Lactobacillus | 5 | -0.0005177 | 0.0007296 | 0.478 |
| phylum Tenericutes | 4 | 0.0001575 | 0.00102 | 0.8773 |
| phylum Proteobacteria | 2 | 0.0006063 | 0.003024 | 0.8411 |
| genus Romboutsia | 1 | -0.001854 | 0.002295 | 0.4192 |
| family Veillonellaceae | 6 | 0.000814 | 0.00121 | 0.5012 |
| family Desulfovibrionaceae | 4 | -0.0006912 | 0.001219 | 0.5708 |
| family Defluviitaleaceae | 2 | -0.0005283 | 0.001249 | 0.6723 |
| family Porphyromonadaceae | 2 | 0.002815 | 0.002859 | 0.3249 |
| genus Ruminiclostridium6 | 2 | 0.0005204 | 0.002468 | 0.833 |
| genus Eggerthella | 3 | -0.0002959 | 0.0008278 | 0.7207 |
| order NB1n | 5 | 0.0005922 | 0.0006082 | 0.3303 |
| genus Christensenellaceae R 7group | 1 | -0.005365 | 0.002597 | 0.03884 |
| class Alphaproteobacteria | 1 | -0.00003964 | 0.001931 | 0.9836 |
| genus Intestinimonas | 4 | 0.001723 | 0.001221 | 0.1584 |
| genus Senegalimassilia | 2 | -0.0003745 | 0.001194 | 0.7538 |
| genus Ruminococcaceae NK4A214 group | 3 | -0.000584 | 0.001765 | 0.7407 |
| genus Ruminococcaceae UCG009 | 5 | -0.0006068 | 0.0007848 | 0.4394 |
| genus Lachnospiraceae NC2004 group | 3 | 0.0009537 | 0.0008813 | 0.2792 |
| family Acidaminococcaceae | 3 | -0.002515 | 0.00128 | 0.04938 |
| class Actinobacteria | 4 | -0.001789 | 0.0009604 | 0.06246 |
| genus Roseburia | 4 | -0.0002443 | 0.001324 | 0.8537 |
| genus Lachnoclostridium | 3 | -0.002859 | 0.00148 | 0.05341 |
| order Gastranaerophilales | 2 | 0.0002084 | 0.001036 | 0.8405 |
| genus Eubacterium hallii group | 4 | 0.001645 | 0.001804 | 0.362 |
| genus Akkermansia | 6 | 0.0001365 | 0.00113 | 0.9039 |
| family Rhodospirillaceae | 3 | -0.001479 | 0.00103 | 0.151 |
| genus Oxalobacter | 3 | 0.0003017 | 0.001387 | 0.8278 |
| family Peptostreptococcaceae | 5 | 0.0001829 | 0.001118 | 0.87 |
| genus Streptococcus | 4 | -0.001909 | 0.001277 | 0.135 |
| genus Coprococcus2 | 3 | 0.001769 | 0.001201 | 0.1408 |
| genus Veillonella | 4 | 0.002212 | 0.0009644 | 0.02179 |
| genus Clostridium sensustricto1 | 1 | -0.0006223 | 0.002367 | 0.7926 |
| class Deltaproteobacteria | 3 | -0.001298 | 0.001407 | 0.3562 |
| order Erysipelotrichales | 4 | 0.0008672 | 0.001267 | 0.4937 |
| class Mollicutes | 4 | 0.0001575 | 0.00102 | 0.8773 |
| genus Eubacterium oxidoreducens group | 2 | 0.000867 | 0.001511 | 0.5661 |
| genus Intestinibacter | 3 | -0.001032 | 0.001179 | 0.3813 |
| order Clostridiales | 4 | 0.003834 | 0.001578 | 0.01511 |
| genus Haemophilus | 3 | -0.0001275 | 0.00104 | 0.9024 |
| genus Lactococcus | 4 | -0.000607 | 0.0006205 | 0.3279 |
| genus Tyzzerella3 | 2 | -0.0004133 | 0.0008619 | 0.6316 |
| genus Oscillospira | 1 | 0.0001813 | 0.002024 | 0.9286 |
| genus Eubacterium xylanophilum group | 3 | -0.001748 | 0.001335 | 0.1906 |
| genus Prevotella7 | 4 | 0.0001956 | 0.0005816 | 0.7367 |
| genus Holdemania | 3 | -0.0002006 | 0.00102 | 0.8441 |
| genus Lachnospiraceae | 1 | 0.0004323 | 0.002373 | 0.8554 |
| family Prevotellaceae | 5 | -0.001865 | 0.001141 | 0.1022 |
| genus Lachnospiraceae NK4A136 group | 3 | -0.001312 | 0.001476 | 0.374 |
| genus Bacteroides | 2 | 0.002257 | 0.001848 | 0.222 |
| class Negativicutes | 4 | -0.001543 | 0.002304 | 0.5033 |
| genus Ruminococcus gauvreauii group | 5 | 0.00002776 | 0.001006 | 0.978 |
| genus Coprococcus1 | 3 | 0.0007996 | 0.001408 | 0.5702 |
| genus Victivallis | 3 | 0.0009215 | 0.0006516 | 0.1573 |
| family Family XI | 5 | 0.001377 | 0.0005041 | 0.006292 |
| genus Odoribacter | 1 | 0.0009197 | 0.002434 | 0.7055 |
| phylum Bacteroidetes | 1 | -0.000426 | 0.002605 | 0.8701 |
| genus Ruminiclostridium5 | 2 | 0.003276 | 0.001827 | 0.07294 |
| genus Barnesiella | 5 | 0.0005398 | 0.001047 | 0.6061 |
| class Erysipelotrichia | 4 | 0.0008672 | 0.001267 | 0.4937 |
| genus Butyricimonas | 4 | -0.0005142 | 0.00102 | 0.6141 |
| genus Methanobrevibacter | 1 | 0.001669 | 0.00119 | 0.1607 |
| genus Ruminococcaceae UCG013 | 2 | -0.0009046 | 0.001665 | 0.587 |
| genus Family XIII AD3011 group | 1 | -0.00005636 | 0.002273 | 0.9802 |
| order Selenomonadales | 4 | -0.001543 | 0.002304 | 0.5033 |
| family Coriobacteriaceae | 3 | 0.000494 | 0.001571 | 0.7531 |
| genus Eubacterium ventriosum group | 4 | -0.0009268 | 0.001222 | 0.4483 |
| genus Ruminococcaceae UCG014 | 3 | -0.001976 | 0.001339 | 0.1399 |
| order Bacillales | 1 | 0.0002801 | 0.001159 | 0.809 |
| family Alcaligenaceae | 5 | 0.001368 | 0.001224 | 0.2637 |
| genus Butyricicoccus | 1 | -0.00634 | 0.002642 | 0.01641 |
| family Lactobacillaceae | 3 | -0.0001995 | 0.0009594 | 0.8353 |
| genus Alloprevotella | 2 | 0.001994 | 0.0007664 | 0.009262 |
| genus Ruminococcaceae UCG011 | 3 | 0.0005438 | 0.0007086 | 0.4428 |
| genus Family XIII UCG001 | 3 | -0.001493 | 0.001284 | 0.2452 |
| order Desulfovibrionales | 4 | -0.0006861 | 0.00122 | 0.5739 |
| genus Allisonella | 4 | -0.0005709 | 0.0005276 | 0.2792 |
| genus Faecalibacterium | 1 | -0.003255 | 0.002578 | 0.2068 |
| genus Terrisporobacter | 1 | 0.001503 | 0.001662 | 0.366 |
| order Rhodospirillales | 3 | -0.0009476 | 0.001032 | 0.3586 |
| genus Olsenella | 4 | -0.00008416 | 0.000624 | 0.8927 |
| family Erysipelotrichaceae | 4 | 0.0008672 | 0.001267 | 0.4937 |
| genus Bifidobacterium | 3 | -0.0008707 | 0.00101 | 0.3887 |
| order Pasteurellales | 2 | 0.000116 | 0.001459 | 0.9366 |
| family Family XIII | 3 | -0.0001589 | 0.001477 | 0.9143 |
| family Clostridiales vadin BB60 group | 4 | 0.0002909 | 0.00102 | 0.7754 |
| genus Eubacterium eligens group | 2 | 0.0001819 | 0.001564 | 0.9074 |
| corder Enterobacteriales | 2 | 0.0003297 | 0.001657 | 0.8422 |
| order Actinomycetales | 1 | 0.0008894 | 0.001784 | 0.618 |
| class Bacteroidia | 2 | -0.001207 | 0.001894 | 0.5238 |
| genus Parasutterella | 7 | -0.0008049 | 0.00107 | 0.4518 |
| genus Subdoligranulum | 3 | -0.0002311 | 0.002579 | 0.9286 |
| order Coriobacteriales | 3 | 0.000494 | 0.001571 | 0.7531 |
| order Methanobacteriales | 1 | 0.001547 | 0.001102 | 0.1607 |
| family Bacteroidaceae | 2 | 0.002257 | 0.001848 | 0.222 |
| family Pasteurellaceae | 2 | 0.000116 | 0.001459 | 0.9366 |
| class Verrucomicrobiae | 6 | 0.0001352 | 0.001128 | 0.9046 |
| genus Erysipelatoclostridium | 6 | 0.0007612 | 0.0007685 | 0.3219 |
| genus Anaerotruncus | 4 | -0.0001397 | 0.001291 | 0.9138 |
| genus Hungatella | 1 | -0.001209 | 0.001234 | 0.3272 |
| genus Ruminiclostridium9 | 2 | 0.004655 | 0.002211 | 0.03522 |
| family Methanobacteriaceae | 1 | 0.001547 | 0.001102 | 0.1607 |
| genus Eubacterium coprostanoligenes group | 3 | 0.002342 | 0.001609 | 0.1455 |
| family Actinomycetaceae | 1 | 0.0008859 | 0.001777 | 0.618 |
| family Rikenellaceae | 4 | 0.001393 | 0.001342 | 0.2992 |
| genus Candidatus Soleaferrea | 3 | 0.0009622 | 0.0008947 | 0.2822 |
| genus Sutterella | 3 | 0.001328 | 0.001321 | 0.315 |
| genus Holdemanella | 5 | 0.1056 | 0.222 | 0.6342 |
| genus Flavonifractor | 4 | 0.1412 | 0.3182 | 0.6573 |
| genus Eubacterium ruminantium group | 5 | 0.001331 | 0.0007698 | 0.08374 |
| genus Paraprevotella | 3 | -0.0003285 | 0.001285 | 0.7982 |
| genus Howardella | 3 | 0.0001382 | 0.0006997 | 0.8434 |
| genus Sellimonas | 5 | -0.0002876 | 0.0004978 | 0.5634 |
| class Gammaproteobacteria | 2 | -0.001517 | 0.001745 | 0.3844 |
| genus Dorea | 1 | -0.00416 | 0.002626 | 0.1131 |
| genus Alistipes | 4 | 0.0005911 | 0.001542 | 0.7016 |
| class Methanobacteria | 1 | 0.001547 | 0.001102 | 0.1607 |
| genus Coprobacter | 2 | -0.0006479 | 0.001189 | 0.5857 |
| genus Prevotella9 | 2 | 0.001994 | 0.001424 | 0.1614 |
| genus Ruminococcaceae UCG002 | 4 | -0.001354 | 0.001464 | 0.3551 |
| class Betaproteobacteria | 4 | 0.001494 | 0.001847 | 0.4184 |
| genus Slackia | 3 | -0.00139 | 0.0008771 | 0.113 |
| genus Coprococcus3 | 3 | 0.0008503 | 0.001423 | 0.55 |
| phylum Actinobacteria | 8 | -0.002067 | 0.0008589 | 0.01609 |
| genus Eubacterium brachy group | 2 | -0.0003967 | 0.0009599 | 0.6794 |
| genus Lachnospira | 1 | -0.003378 | 0.002557 | 0.1864 |
| genus Phascolarctobacterium | 2 | 0.001534 | 0.001718 | 0.3721 |
| genus Bilophila | 2 | 0.001534 | 0.001718 | 0.3721 |
| family Streptococcaceae | 3 | 0.001504 | 0.002042 | 0.4616 |
| order Victivallales | 1 | -0.002922 | 0.001354 | 0.03095 |
| genus Ruminococcus torques group | 2 | -0.00149 | 0.001692 | 0.3787 |
| genus Escherichia Shigella | 2 | -0.0005637 | 0.00156 | 0.7178 |
| genus Lachnospiraceae UCG004 | 6 | -0.001176 | 0.0009739 | 0.2273 |
| genus Rikenellaceae RC9 gut group | 3 | -0.0009105 | 0.0006825 | 0.1822 |
| genus Lachnospiraceae UCG010 | 2 | 0.0003366 | 0.001595 | 0.8329 |
| genus Oscillibacter | 6 | 0.00133 | 0.000891 | 0.1356 |
| order Bacteroidales | 2 | -0.001207 | 0.001894 | 0.5238 |
| class Coriobacteriia | 3 | 0.000494 | 0.001571 | 0.7531 |
