# Supplementary material for: The causal correlation between gut microbiota abundance and pathogenesis of cervical cancer: a bidirectional mendelian randomization study
Source: Front Microbiol. 2024 Feb 14;15:1336101. doi: 10.3389/fmicb.2024.1336101 (PMC10901247; doi:10.3389/fmicb.2024.1336101)
Supplement: Supplementary file 2 [file Table_2.docx]

Table S2. Primary causality of gut microbiota abundance on the risk of cervical cancer

| exposure | n SNP | IVW/Wald ratio | | | MR Egger | | | Weighted median | | | horizontal pleiotropy | | | Heterogeneity | | Causal direction | | |
| --- | --- | --- | --- | --- | --- | --- | --- | --- | --- | --- | --- | --- | --- | --- | --- | --- | --- | --- |
|  |  | b | SE | P-val | b | SE | P-val | b | SE | P-val | ERI | SE | P-val | Q | P-val | WEIE | WEIO | P-val |
| class Clostridia | 4 | 0.00382 | 0.001575 | 0.01526 | -0.01513 | 0.01726 | 0.4732 | 0.005782 | 0.002841 | 0.1346 | 0.0011 | 0.00097 | 0.385 | 4.23 | 0.4014 | 0.0065 | 2.7e-05 | 7.16e-19 |
| class Lentisphaeria | 1 | -0.002922 | 0.001354 | 0.03095 | - | - | - | - | - | - | - | - | - | - | - | 0.0031 | 1.3e-05 | 1.04e-09 |
| family Acidaminococcaceae | 3 | -0.002515 | 0.00128 | 0.03575 | -0.008064 | 0.01902 | 0.7447 | -0.00294 | 0.0017 | 0.08372 | 0.00039 | 0.0013 | 0.819 | 1.831 | 0.4002 | 0.0061 | 1.4e-05 | 1.4e-05 |
| family Family XI | 5 | 0.001377 | 0.0005041 | 0.006292 | 0.004768 | 0.005322 | 0.4364 | 0.001596 | 0.0006636 | 0.01617 | -0.00043 | 0.00067 | 0.568 | 0.4067 | 0.9389 | 0.0077 | 1.8e-05 | 5.99e-23 |
| genus Alloprevotella | 2 | 0.001994 | 0.001994 | 0.009262 | - | - | - | - | - | - | - | - | - | 0.002765 | 0.9581 | 0.0015 | 1.3e-05 | 3.6e-05 |
| genus Christensenellaceae R7 group | 1 | -0.005365 | 0.002597 | 0.038 | - | - | - | - | - | - | - | - | - | - | - | 0.0032 | 1.9e-05 | 7.02e-10 |
| genus Marvinbryantia | 3 | -0.002662 | 0.001197 | 0.02618 | -0.004067 | 0.01263 | 0.8017 | -0.002782 | 0.00157 | 0.0765 | 9.9e-05 | 0.00088 | 0.929 | 1.736 | 0.4197 | 0.0064 | 1.5e-05 | 1.73e-19 |
| genus Ruminiclostridium 9 | 2 | 0.004655 | 0.004655 | 0.03522 | - | - | - | - | - | - | - | - | - | 1.354 | 0.244 | 0.0063 | 2.7e-05 | 1.54e-18 |
| order Clostridiales | 4 | 0.003834 | 0.001578 | 0.01511 | -0.01464 | 0.01734 | 0.4875 | 0.003922 | 0.001783 | 0.001783 | 0.001 | 0.00097 | 0.397 | 4.221 | 0.2385 | 0.0064 | 2.7e-05 | 9.38e-19 |
| order Victivallales | 1 | -0.002922 | 0.001354 | 0.0309 | - | - | - | - | - | - | - | - | - | - | - | 0.0031 | 1.3e-05 | 1.04e-09 |
| phylum Actinobacteria | 8 | -0.002067 | 0.0008589 | 0.01609 | -0.001991 | 0.004354 | 0.6635 | -0.002253 | 0.00113 | 0.00113 | -4.4e-06 | 0.00025 | 0.986 | 1.116 | 0.9927 | 0.019 | 1.9e-05 | 3.8e-55 |
| phylum Lentisphaerae | 2 | -0.002147 | 0.0009549 | 0.02456 | - | - | - | - | - | - | - | - | - | 0.5996 | 0.4387 | 0.0046 | 1.5e-05 | 3.86e-14 |

WEIE=Variance explained in exposure, WEIO=Variance explained in outcome, SE=Standard error, ERI=Egger regression intercept
