# Supplementary material for: The causal correlation between gut microbiota abundance and pathogenesis of cervical cancer: a bidirectional mendelian randomization study
Source: Front Microbiol. 2024 Feb 14;15:1336101. doi: 10.3389/fmicb.2024.1336101 (PMC10901247; doi:10.3389/fmicb.2024.1336101)
Supplement: Supplementary file 3 [file Table_3.docx]

Table S3. Verified causality of gut microbiota abundance on the risk of cervical cancer

| exposure | n SNP | IVW/Wald ratio | | | MR Egger | | | Weighted median | | | Horizontal pleiotropy | | | Heterogeneity | | Causal direction | | |
| --- | --- | --- | --- | --- | --- | --- | --- | --- | --- | --- | --- | --- | --- | --- | --- | --- | --- | --- |
|  |  | b | SE | P-val | b | SE | P-val | b | SE | P-val | ERI | SE | P-val | Q | P-val | WEIE | WEIO | P-val |
| class Methanobacteria | 4 | 0.001967 | 0.0007179 | 0.01526 | 0.0006656 | 0.002757 | 0.8317 | 0.001766 | 0.0008361 | 0.03462 | 0.00023 | 0.00047 | 0.673 | 0.24 | 0.9709 | 0.0067 | 3.9e-05 | 2.79e-18 |
| family Actinomycetaceae | 2 | 0.002954 | 0.001324 | 0.02572 | - | - | - | - | - | - | - | - | - | 0.005385 | 0.9415 | 0.0031 | 2.5e-05 | 4.97e-09 |
| family Methanobacteriaceae | 4 | 0.001967 | 0.0007179 | 0.006149 | 0.0006656 | 0.002757 | 0.8317 | 0.001766 | 0.0008618 | 0.04039 | 0.00023 | 0.00047 | 0.673 | 0.24 | 0.9709 | 0.0067 | 3.9e-05 | 2.79e-18 |
| family Streptococcaceae | 7 | -0.003037 | 0.001172 | 0.009596 | -0.005603 | 0.004427 | 0.2614 | -0.002966 | 0.001526 | 0.05193 | 0.00019 | 0.00032 | 0.574 | 2.161 | 0.9043 | 0.015 | 4.9e-05 | 1.27e-40 |
| genus Dialister | 2 | -0.003885 | 0.001909 | 0.04181 | - | - | - | - | - | - | - | - | - | 0.08058 | 0.7765 | 0.0032 | 2.1e-05 | 1.73e-09 |
| genus Lachnospiraceae UCG 010 | 5 | 0.002696 | 0.001247 | 0.03066 | 0.0002074 | 0.0051 | 0.9701 | 0.003654 | 0.001655 | 0.02726 | 0.00021 | 0.00042 | 0.649 | 2.619 | 0.6235 | 0.008 | 3.7e-05 | 5.08e-22 |
| genus Methanobrevibacter | 3 | 0.00202 | 0.000856 | 0.01831 | 0.0005035 | 0.0005035 | 0.882 | 0.001758 | 0.001098 | 0.1093 | 0.00025 | 0.00043 | 0.658 | 0.3608 | 0.8349 | 0.0047 | 3e-05 | 3.37e-13 |
| order Actinomycetales | 2 | 0.00296 | 0.001327 | 0.02573 | - | - | - | - | - | - | - | - | - | 0.005825 | 0.9392 | 0.9392 | 2.5e-05 | 5.46e-09 |
| order Methanobacteriales | 4 | 0.001967 | 0.0007179 | 0.006149 | 0.0006656 | 0.002757 | 0.8317 | 0.001766 | 0.0008537 | 0.03852 | 0.00023 | 0.00047 | 0.673 | 0.24 | 0.9709 | 0.0067 | 3.9e-05 | 2.79e-18 |
| phylum Bacteroidetes | 6 | -0.08996 | 0.02713 | 0.0009156 | -0.1332 | 0.06498 | 0.1097 | -0.09648 | -0.09648 | 0.005602 | 0.004 | 0.0055 | 0.504 | 2.473 | 0.7805 | 0.013 | 8e-05 | 1.17e-34 |

WEIE=Variance explained in exposure, WEIO=Variance explained in outcome, SE=Standard error, ERI=Egger regression intercept
