# Supplementary material for: The causal correlation between gut microbiota abundance and pathogenesis of cervical cancer: a bidirectional mendelian randomization study
Source: Front Microbiol. 2024 Feb 14;15:1336101. doi: 10.3389/fmicb.2024.1336101 (PMC10901247; doi:10.3389/fmicb.2024.1336101)
Supplement: Supplementary file 4 [file Table_4.docx]

Table S4. Primary causality of CC on gut microbiota abundance

| Exposure | n SNP | IVW/Wald ratio | | | MR Egger | | | Weighted median | | | Horizontal pleiotropy | | | Heterogeneity | | Causal direction | | |
| --- | --- | --- | --- | --- | --- | --- | --- | --- | --- | --- | --- | --- | --- | --- | --- | --- | --- | --- |
|  |  | b | SE | P-val | b | SE | P-val | b | SE | P-val | ERI | SE | P-val | Q | P-val | WEIE | WEIO | P-val |
| class Verrucomicrobiae | 6 | 17.12 | 7.958 | 0.03145 | 53.35 | 66.37 | 0.8317 | 14.49 | 9.625 | 0.1322 | -0.027 | 0.05 | 0.612 | 1.718 | 0.8866 | 4e-04 | 0.00043 | 0.928 |
| family Defluviitaleaceae | 6 | -20.5 | 9.63 | 0.03323 | 43.99 | 81.19 | 0.6167 | -18.55 | 11.87 | 0.1181 | -0.048 | 0.06 | 0.469 | 2.044 | 0.843 | 4e-04 | 0.00059 | 0.605 |
| family Verrucomicrobiaceae | 6 | 17.12 | 7.958 | 0.03149 | 53.36 | 66.37 | 0.4665 | 14.47 | 9.791 | 0.1395 | -0.027 | 0.05 | 0.612 | 1.707 | 0.888 | 4e-04 | 0.00043 | 0.929 |
| genus Akkermansia | 6 | 17.14 | 7.958 | 0.03125 | 54.39 | 66.37 | 0.4585 | 17.14 | 7.958 | 0.03125 | -0.028 | 0.05 | 0.602 | 1.726 | 0.8856 | 4e-04 | 0.00043 | 0.925 |
| genus Barnesiella | 6 | -20.76 | 7.977 | 0.009246 | -55.35 | 72.41 | 0.4872 | -28.06 | 9.301 | 0.002549 | 0.026 | 0.054 | 0.656 | 5.986 | 0.3076 | 4e-04 | 0.001 | 0.17 |
| genus Defluviitaleaceae UCG011 | 6 | -18.96 | 9.642 | 0.04928 | 60.47 | 81.28 | 0.4982 | -17.51 | 12.55 | 0.1629 | -0.06 | 0.061 | 0.381 | 2.547 | 0.769 | 4e-04 | 0.00059 | 0.608 |
| genus Lachnospiraceae UCG001 | 6 | -19.86 | 8.597 | 0.02088 | -60.59 | 71.6 | 0.4451 | -15.76 | 11.19 | 0.1093 | 0.031 | 0.053 | 0.597 | 4.079 | 0.5381 | 4e-04 | 0.00084 | 0.285 |
| genus Ruminiclostridium5 | 6 | 16.33 | 6.596 | 0.01329 | -7.143 | 55.04 | 0.903 | 16.29 | 8.22 | 0.04749 | 4e-04 | 0.00061 | 0.575 | 1.915 | 0.8607 | 4e-04 | 0.00061 | 0.575 |
| order Verrucomicrobiales | 6 | 17.12 | 7.958 | 0.03145 | 53.35 | 66.37 | 0.4665 | 14.49 | 9.75 | 0.1372 | -0.027 | 0.05 | 0.612 | 1.718 | 0.8866 | 4e-04 | 0.00043 | 0.928 |
| phylum Verrucomicrobia | 6 | 18.41 | 7.796 | 0.01823 | 35.29 | 65.08 | 0.6164 | 19.92 | 9.338 | 9.338 | -0.013 | 0.049 | 0.807 | 0.5771 | 0.989 | 4e-04 | 0.00043 | 0.923 |

WEIE=Variance explained in exposure, WEIO=Variance explained in outcome, SE=Standard error, ERI=Egger regression intercept
