# Supplementary material for: The causal correlation between gut microbiota abundance and pathogenesis of cervical cancer: a bidirectional mendelian randomization study
Source: Front Microbiol. 2024 Feb 14;15:1336101. doi: 10.3389/fmicb.2024.1336101 (PMC10901247; doi:10.3389/fmicb.2024.1336101)
Supplement: Supplementary file 5 [file Table_5.docx]

Table S5. Verified causality of CC on gut microbiota abundance

| Exposure | n SNP | IVW/Wald ratio | | | MR Egger | | | Weighted median | | | Horizontal pleiotropy | | | Heterogeneity | | Causal direction | | |
| --- | --- | --- | --- | --- | --- | --- | --- | --- | --- | --- | --- | --- | --- | --- | --- | --- | --- | --- |
|  |  | b | SE | P-val | b | SE | P-val | b | SE | P-val | ERI | SE | P-val | Q | P-val | WEIE | WEIO | P-val |
| genus Alloprevotella | 3 | -0.1618 | 0.07299 | 0.02661 | 0.3501 | 0.5614 | 0.6451 | -0.1612 | 0.0776 | 0.03775 | -0.17 | 0.19 | 0.526 | 3.562 | 0.1685 | - | - | - |
| genus Eisenbergiella | 5 | 22.22 | 10.62 | 0.03636 | 17.52 | 58.37 | 0.7837 | 21.63 | 13.05 | 13.05 | 0.0055 | 0.067 | 0.94 | 0.8429 | 0.9326 | 0.00078 | 0.00042 | 0.382 |
| genus  Eubacteriumnodatum group | 5 | -40.41 | 18.45 | 0.02848 | -60.74 | 116.2 | 0.6372 | -54.07 | 20.42 | 0.008104 | 0.024 | 0.13 | 0.87 | 7.137 | 0.128 | 0.00078 | 0.0014 | 0.305 |
| genus Phascolarctobacterium | 5 | -15.58 | 7.512 | 0.0381 | 1.157 | 43.5 | 0.9804 | -15.58 | 7.512 | 0.0381 | -0.019 | 0.05 | 0.722 | 3.523 | 0.4744 | 0.00078 | 0.00087 | 0.858 |
| phylum Euryarchaeota | 5 | 31.98 | 12.96 | 0.01365 | -49.87 | 70.02 | 0.5277 | 45.76 | 17.15 | 0.00764 | 0.095 | 0.08 | 0.32 | 4.068 | 0.3968 | 0.00078 | 0.001 | 0.619 |

WEIE=Variance explained in exposure, WEIO=Variance explained in outcome, SE=Standard error, ERI=Egger regression intercept
