# Supplementary figures and images for: The causal correlation between gut microbiota abundance and pathogenesis of cervical cancer: a bidirectional mendelian randomization study
Source: Front Microbiol. 2024 Feb 14;15:1336101. doi: 10.3389/fmicb.2024.1336101 (PMC10901247; doi:10.3389/fmicb.2024.1336101)

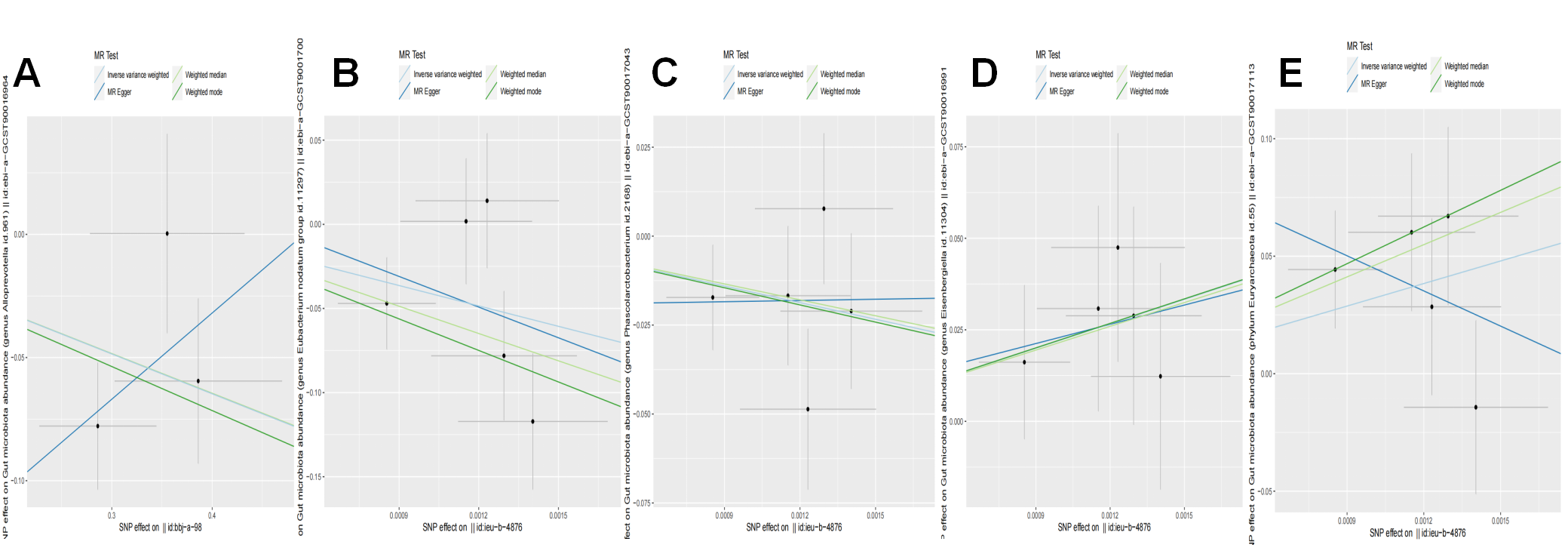

Supplement: Supplementary file 6 [file Image_1.TIF]
